# Effect of statin use on outcome of symptomatic cholelithiasis: a case-control study

**DOI:** 10.1186/1471-230X-14-119

**Published:** 2014-07-03

**Authors:** Jukka Pulkkinen, Matti Eskelinen, Vesa Kiviniemi, Tuukka Kotilainen, Markus Pöyhönen, Lasse Kilpeläinen, Pirjo Käkelä, Helena Kastarinen, Hannu Paajanen

**Affiliations:** 1 Department of Surgery, Kuopio University Hospital, P.O. Box 100, FI-70029 KYS Kuopio, Finland; 2 School of Medicine, University of Eastern Finland, P.O. Box 1627, FI-70211 Kuopio, Finland; 3 Finnish Medicines Agency, P.O. Box 55, FI-00034 FIMEA Kuopio, Finland

**Keywords:** Cholelithiasis, Statins, Cholecystectomy

## Abstract

**Background:**

Statins can modify bile cholesterol and, thus, the formation of gallstones. We examined whether statin use also modifies the severity of symptomatic gallstone disease and its treatment.

**Methods:**

A total of 1,140 consecutive patients with symptomatic gallstone disease were recruited during 2008-2010 at Kuopio university hospital, Finland. Case-control analysis matched the patients using (n = 272) or not using (n = 272) statins by age and sex. The baseline characteristics of the patients, need and type of surgical treatment, duration of operation, perioperative bleeding, postoperative complications and overall mortality rate were compared statistically between the study groups.

**Results:**

Morbidity and subsequent polypharmacy occurred more frequently among the patients with statins compared to the patients without statins. There were no significant differences between the statin users and non-users regarding surgical treatment (open vs. laparoscopic cholecystectomy). The mean operation time for laparoscopic cholecystectomy was 10% shorter for the patients with statin use than for the patients without. In addition, there was a non-significant tendency for statin users to bleed less during laparoscopic operations than the non-users. There were no differences in other procedure-related parameters (e.g., operation urgency, conversions, choledochotomies, complications and mortality) in patients with or without statins.

**Conclusions:**

Compared to no treatment, statin treatment was associated with a shorter operation time for laparoscopy cholecystectomy. Other surgical outcome parameters were similar in patients with or without statins, although statin users had more polypharmacy and circulatory illnesses than non-users.

## Background

Gallstone disease and cholesterol-lowering statin medications are common in Western countries, and they have a high economic impact [1]. Symptomatic gallstones are one of the leading causes of inpatient admissions in general surgery [2]. The prevalence of gallstone disease is increasing because of worldwide epidemics of obesity, insulin resistance and aging. In Western populations, the majority of gallstones formed in the gallbladder consist mainly of cholesterol, as they are formed from cholesterol-supersaturated bile [3]. Cholesterol gallstones account for 80-95% of the gallstones found during cholecystectomy [4,5].

Hypercholesterolemia is an important risk factor for atherosclerosis, which has been the most common cause of death in developed countries. Statins are used to treat dyslipidemia, which prevents cardiovascular or cerebrovascular events. Statins are among the most widely used medications in the Western world [6,7]. As in other Western societies, statin use in Finland has increased exponentially: There has been an 11-fold increase in statin use between 1995 and 2005 [8]. According to the reimbursement register data of the Social Insurance Institute of Finland (population 5.2 million), approximately 660,000 individuals purchased statins in 2012, which is 12% of population.

The relationship between statins and symptomatic gallstone disease is still more or less conflicting, although several studies have reported a significant reduction in the incidence of symptomatic gallstones disease in patients using statin therapy [9]. The most consistent evidence is that bile is desaturated of cholesterol after the long-term administration of statins [10,11]. These observations suggest that statins may reduce the size of cholesterol particles and, subsequently, prohibit gallstone formation and reduce the size of gallstones; therefore, they may also affect the course of symptomatic gallstone disease. The effect of statin therapy on the course and outcome of cholecystectomy is unknown.

We conducted a case control study to assess the influence of statins on complicated gallstone disease. Based on the effect of statins on gallstone formation, we hypothesized that statin use would be associated with less severe acute gallbladder inflammation, fewer stones in the common bile duct and, furthermore, fewer complications.

## Methods

A total of 1,140 consecutive patients with symptomatic gallstones were admitted to Kuopio University Hospital, Finland, from 2008-2010. The hospital serves an area of 243,000 inhabitants. A total of 272 of 1,140 patients with symptomatic cholelithiasis underwent statin therapy. The age- and sex-matched controls (n = 272) were randomly selected from the cohort of patients without statin therapy. Patient data were obtained from the hospital records using the following discharge diagnoses (ICD 10): K80 (cholelithiasis), K81 (cholecystitis), K82 (other disease of gallbladder) or K83 (other diseases of biliary tract). The imaging study results (i.e., computed tomography, abdominal ultrasound, magnetic resonance cholangiography) were analyzed. Additional work-ups included a detailed clinical history with records of recent infectious diseases, abdominal traumas, surgery, and endoscopic retrograde cholangiopancreatography (ERCP), as well as data concerning comorbidities and medicine use on arrival. The laboratory data included serum calcium, lipids, liver enzymes and C-reactive protein measurements. The duration of hospital stay, need for intensive care treatment and one-month hospital mortality (2008-2010) were recorded. Data concerning the treatment type (e.g., conservative, laparoscopic, open surgery, conversions, cholecystostomy, and choledochotomies), surgical durations, elective vs. emergency operations, complications, bleeding and number of re-operations were collected from the patients records.

Surgery was performed in both study groups using the same operative principles and by the same group of surgeons. There were no between-group differences in the preoperative or perioperative treatments or procedures. Emergency cholecystectomy was performed if ultrasonography or clinical examination and laboratory parameters revealed signs of cholecystitis in and if the patient was suitable for operation, according to the American Society of Anesthesiologists physical status classification system. If possible, perioperative cholangiography was always performed in acute cholecystitis. Cholecystectomy was primarily performed laparoscopically. In patients with very old age and severe comorbidities, percutaneous cholecystostomy was performed. In patients with long histories of cholecystitis symptoms, the treatment was first conservative, and elective laparoscopy was performed later. According to the Finnish legislation, no ethical assessment or approval is mandatory for a register-based study. As this is a retrospective register study, informed consent from the patients was not needed.

Data were analyzed using IBM SPSS (Statistical Package for the Social Sciences) Statistics 21.0 (IBM Corp. Released 2012. IBM SPSS Statistics for Windows, Version 21.0. Armonk, NY: IBM Corp. USA), and the statistical analyses were performed using independent samples t-tests, Mann-Whitney U-tests and χ^2^ tests, depending on the distribution of the outcome and distributional assumptions. Adjusted analyses of outcomes were not conducted because of the relatively small number of events in various outcomes of interest. P values less than 0.05 were considered to be statistically significant.

## Results

A total of 1140 admissions of the patients with symptomatic cholelithiasis were recorded at our hospital between 2008 and 2010. There were 715 females and 425 males (63% and 37%, respectively). Some 272 patients used statins (24% of the whole study population), 132 females and 140 males (17.6% and 32.9%, respectively), (Table 1). The patients using statin therapy were, on average, older, had a greater incidence of type II diabetes and cardiovascular diseases and were more frequently male than the patients without statin therapy (data not shown).

**Table 1:** Demographic data of statin users (n = 272) and non-statin users (n = 272) patients with gallstones

	**Statin users (n = 272)**	**No statin (n = 272)**	**P-value**
Females/males	132/140 (48%/52%)	132/140 (48%/52%)	1.000
Age (years)	69 ± 11 (36-93)	69 ± 11 (36-93)	0.786
Body mass index (Kg/m^2^)	29 ± 4.2	28 ± 4.9	0.499
Use of statins			
Simvastatin	73%	199	
Atorvastatiini	13%	36	
Rosuvastatin	6%	15	
Fluvastatin	4%	11	
Pravastatin	2%	5	
Lovastatin	1%	3	
Other^1^	1%	3	
Comorbid condition			
Hypertension	161 (59%)	115 (42%)	< 0.001
Diabetes	48(18%)	38 (14%)	0.290
Coronary artery disease/			
Coronary heart failure/			
Valvular	151 (56%)	71 (26%)	< 0.001
Neurological	50 (18%)	48 (18%)	0.456
Psychiatric	16 (6%)	22 (8%)	0.401
Pulmonary	32 (12%)	37 (14%)	0.303
Rheumatoid	12 (4%)	12 (4%)	1.000
Malignancy	26 (10%)	33 (12%)	0.408
Number of medication			
No	0	75 (28%)	
1-3 drug	44 (16%)	88 (32%)	
> 4 drugs	228 (84%)	109 (40%)	

^1^Means combination of statin with other cholesterol-lowering drugs (fenofibrate, ezetimibe).

Values are presented as number (percentage) or mean (SD, min-max), as appropriate.

### Statin users vs. their matched controls

In the case control comparison, hypertension and cardiac diseases were observed more frequently among the statin users compared with the non-users (Table 1). On the contrary, the prevalence of diabetes, neurological, psychiatric, and pulmonary diseases, as well as malignancy, were similar in both study groups. The mean BMI was similar between the groups. In the statin group, simvastatin constituted 73% and atorvastatin 13% of all statins (Table 1).

We also calculated the number of regularly used drugs in both groups. Polypharmacy (>4 medicines) was observed in more than 80% of the patients in the statin group and in 48% of the patients in the no-statin group (p < 0.001). In the non-use group, 28% of the patients were not using any medicines upon admission (data not shown).

We did not observe any statistically significant between-group differences in any of the assessed laboratory parameters, including LDL cholesterol. The mean C-reactive protein concentration was 112 mg/l in the statin group and 113 mg/l in the no-statin group (Table 2).

**Table 2:** Laboratory data of study groups

	**Statin use (n = 272)**	**No statin (n = 272)**	**p-value**
P-Amyl U/l	76 (166)	113 (478)	0.873
P-Afos U/l	134 (148)	131 (132)	0.924
P-Bil umol/l	24 (31)	30 (42)	0.171
ALAT U/l	89 (141)	108 (181)	0.639
fP-Kol-LDL mmol/l	2.2 (1.0)	2.4 (1.0)	0.622
fP-TRIG mmol/l	1.6 (0.9)	1.5 (0.8)	0.805
CRP mg/l	112 (100)	113 (111)	0.604

Values are expressed as mean (SD).

p-values are indicated using the Mann-Whitney U-test.

Approximately 27% of patients in the statin group and 25% of patients in the non-statin group were treated conservatively (Table 3). Approximately 68% of all operations were elective, and 32% were emergency procedures. Three quarters of the operations were started with laparoscopy. The mean conversion rate to open surgery was 13%. One patient in the non-statin group was re-operated two times: first because of bile leak and again because of abscesses. We found no significant differences between the statin users and non-users regarding the treatment types (elective/emergency, laparoscopic/open) or in the number of conversions or ERCP procedures (Table 3). Furthermore, there were no significant differences in the rate of complications, need for intensive care treatment or hospital mortality. Interestingly, the hospital mortality rates were similar among the statin users and the non-users (Table 3).

**Table 3:** Treatment and outcome of patients with cholelithiasis

	**Statin use (n = 272)**	**No statin (n = 272)**	**p-value**
Overall mortality	12 (4%)	19 (7%)	0.195
ICU treatment (N patients)	6 (2%)	3 (1%)	0.348
Mean hospital stay, days	4.3 ± 9.9	3.9 ± 4.7	0.730^1^
Treatment type			
Conservative/Surgical	73/199 (27/73%)	68/204 (25/75%)	0.625
Elective/emergency operation	138/61 (69/31%)	136/68 (67/33%)	0.769
Laparoscopic	131 (65%)	130 (64%)	0.659
Open surgery	47 (24%)	46 (23%)	0.799
Conversion	13 (7%)	17 (8%)	0.491
Cholecystostoma	8 (4%)	11 (5%)	0.516
Reoperations	6 (3.0%)	3 (1.5%)	*0.211*
Choledochotomy	13 (7%)	20 (10%)	0.197
Choledocholithiasis	9 (5%)	20 (10%)	0.104
Complications (operated)	27 (13.6%)	32 (15.7%)	0.733
Urological	5 (2.5%)	7 (3.4%)	
Peritonitis	3 (1.5%)	3 (1.5%)	
Bleeding	4 (2.0%)	3 (1.5%)	
Bile leak	3 (1.5%)	1 (0.5%)	
Other abscess	1 (0.5%)	1 (0.5%)	
Heart complication	1 (0.5%)	1 (0.5%)	
Lung complication	1 (0.5%)	0	
Other	9 (4.5%)	16 (7.8%)	
ERCP (all)	31 (11%)	34 (13%)	0.595

Values are shown as number (percentage) or mean (SD) unless otherwise indicated.

^1^Indicates the Mann-Whitney U-test.

In both study groups, open surgery was more often conducted as an emergency procedure compared with laparoscopic surgery (Table 4).

**Table 4:** Outcome of cholelithiasis in open and laparoscopic surgery

	**Statin use (n = 272)**	**No statin (n = 272)**	**p-value**
Surgery (laparoscopic and open)	N = 178	N = 176	
Laparoscopic	131 (74%)	130 (74%)	
Elective/Emergency	116/15 (89%/11%)	109/21 (84%/16%)	0.271
Mean operation time	71.4 ± 31.5	80.81 ± 39.2	0.042
Mean bleeding	20.8 ± 79.2	32.6 ± 88.1	0.070
Bleeding over 100 ml	7 (5%)	14 (11%)	0.099
Mean hospital stay, days	3.1 ± 13.5	2.2 ± 3.4	0.600^1^
Proportion of patients			
with more than 3 hospital days	42 (32%)	46 (35%)	0.602
Proportion of patients			
with more than 5 hospital days	13 (10%)	10 (8%)	0.525
Open surgery (conversions excl.)	47 (26%)	46 (26%)	
Elective/Emergency	14/33 (30%/70%)	15/31 (33%/67%)	0.769
Mean operation time	92.9 ± 38.1	104.8 ± 49.2	0.248
Mean bleeding	208.2 ± 275.1	229.5 ± 276.0	0.332
Bleeding over 100 ml	20 (43%)	18 (39%)	0.960
Mean hospital stay	6.1 ± 4.2	6.4 ± 6.5	0.788
Proportion of patients with more than 5 hospital days	28 (60%)	25 (54%)	0.611

Data are shown as mean (SD) or (percentage) unless otherwise indicated.

^1^Indicates the Mann-Whitney U-test.

Interestingly, the mean time for laparoscopic operations was 9.4 minutes shorter in the statin group compared with the control group (p = 0.042). In addition, the time needed for open surgery was shorter in the statin group compared with the non-statin group, although this difference was not statistically significant. Likewise, there was a non-significant trend of increased bleeding among the non-statin group.

Logistic regression analysis was used to adjust for age, gender and outcomes. The results did not differ from the unadjusted results presented in Table 4. Overall, the number of patients having outcomes was too small to conduct more extensive adjusted analyses (i.e., adjusting for co-morbidities). All open and converted patients remained, on average, three days longer in the hospital than the laparoscopic patients (mean 5.8 ± 5.0 and 2.6 ± 9.9, respectively, P < 0.001). There were no between-group differences regarding the hospitalization time (Table 4).

## Discussion

The main finding of the present study was that the patients using statins did not have worse outcomes after cholecystectomy than the non-users, although the statin users were older, had polypharmacy and demonstrated more comorbidities than the non-users. Statin therapy was also associated with a shorter laparoscopic operation time. There was also a trend of other benefits from statin use for surgical outcomes, such as less bleeding. These findings are of clinical importance, as one would anticipate that statin users would have more post-surgical complications than non-users. A well-conducted prospective randomized study to show the possible benefits of statins in cholecystectomy is warranted.

The risk of developing symptoms or complications related to gallstones is approximately 1–4% per year [12,13]. The known risk factors for gallstones are female gender, increasing age, obesity, metabolic syndrome, rapid weight loss and gallbladder stasis [14,15]. The most important complications of the gallstone disease are biliary pancreatitis, cholecystitis and cholangitis. Festi et al. showed that during follow-up, approximately 80% of gallstones remain asymptomatic, 10% develop mild symptoms, and 10% develop severe symptoms leading to cholecystectomy [16]. The development of the symptomatic gallstones is associated with obesity and alcohol use, likely because of their effects on serum lipids [17]. It is presently unknown whether statin therapy would transform asymptomatic cholelithiasis to symptomatic.

The use of statins and other lipid-lowering drugs has been greatly increasing in Finland in recent years [8]. This increase has occurred particularly among elderly patients with hypercholesterolemia [18]. Prior to beginning our study, we anticipated that statin users would have less severe acute gallbladder inflammation, fewer stones in the common bile duct and more frequent laparoscopic cholecystectomy than non-users; this hypothesis proved wrong. Statins reduce the bile cholesterol content, which may theoretically reduce the risk of developing micro-gallstones or sludge. In the present study, the 1-year incidence of symptomatic cholelithiasis was approximately 0.22% among statin users and 0.14% among non-statin users (these calculations are based on reimbursement register data, data not shown).

Bodmer et al. performed a case control study using a UK-based database of approximately 5 million patients to investigate statins and the reduced risk of gallstone disease. In this large observational study, a lower incidence of cholecystectomy was noted in patients taking statins [3]. In addition, Tsai et al. performed a large retrospective cohort study among 2479 American women who had histories of gallstones. The authors found that statin use was associated with a reduced risk of cholecystectomy [10]. Smith et al. observed that simvastatin (20 mg/day) decreased plasma and biliary cholesterol levels by reducing cholesterol synthesis after 3 weeks of medication [19]. A long-term study in prairie dogs found that lovastatin alters biliary lipid composition and dissolves gallstones [20]. However, most human studies did not find that statin monotherapy would lead to the complete dissolution of gallstones [11]. In accordance with this finding, the need of surgery of any type or the cholelithiasis outcomes did not differ between the statin and non-statin groups, which contrasted with some previous studies. In a Taiwanese study, the investigators did not find a beneficial association between statin use and gallstone disease [21]. However, in East Asia, there is a lower prevalence of gallstones, and the stones primarily have a brown pigment, which indicates that they are comprised mainly of calcium [1].

Statins were not associated with a lower total serum bilirubin level in our study. In 2011, Ong et al. published a contrasting finding. They investigated whether statins in routine use increase the total bilirubin levels in subjects at high cardiovascular risk; but unexpectedly, the authors found that statin use was associated with lower total bilirubin levels. They suggested that this result could be explained, at least partially, by the effect of statins on glycemia and LDL cholesterol [22].

The clinical outcomes did not differ significantly between the study groups, except for the laparoscopic operation time. A shorter laparoscopic operation time was unexpected, as these patients more often have heart disease or hypertension. Conversely, the patients with cardiac disorders used more medications, thereby affecting the bleeding cascade, which would predispose them to bleeding complications. On average, more bleeding was observed among non-users compared with users during laparoscopic operations, but the difference remained statistically insignificant with a p value of 0.07. However, this finding is in line with the shorter operation time among statin users. Possible mechanisms behind these findings may include smaller size of cholesterol particles and gallstones. More research with larger sample sizes is warranted to confirm these findings. The two-year mortality rate (the study time mortality) was also similar among the statin users and non-users. Four patients died in the hospital. Longer observation time mortality was not studied in this study.

The mechanism by which statins might shorten the operation time or diminish bleeding remains unknown and requires further study. There were no differences in the perioperative procedures between the groups, and the same surgical team was in charge of their treatment. There is evidence that statins might have relevant anti-inflammatory effects independent of their lipid lowering ability [23].

The factors that contribute to supersaturating cholesterol in bile and to the formation of cholesterol gallstones are multifactor. Major sources of cholesterol for biliary secretion are the intestine, liver hepatocytes and circulating lipoproteins. Gallbladder hypomotility, destabilization of bile by kinetic protein factors, abnormal mucins and genetic factors also play roles in cholesterol gallstone disease [24]. Many other factors are associated with cholesterol gallstones formation, such as dietary and life style factors and associated conditions (obesity, estrogen therapy, metabolic syndrome, etc.).

Our study had some limitations. Because this study was a retrospective analysis, we could not determine whether the gallstone formation developed before the statin medication was administered. Gallstone formation occurs over a long time period. Furthermore, we do not have accurate data on the patients’ adherence to statin use nor on the duration of the statin treatment in individual patients. Most gallstones are asymptomatic, and the patient does not require a cholecystectomy or hospital admission [25]. In this study, all patients were hospitalized because they had gallstone-related symptoms or complications. We do not have data from the patients with asymptomatic gallstone disease.

## Conclusion

The patients using statins did not have worse outcomes after cholecystectomy than the non-users.

We found that statins might shorten laparoscopic surgery time in patients who have symptomatic cholelithiasis. Statin treatment may also be associated with less bleeding during laparoscopic operations. The beneficial effects of statins were observed, although statin use was associated with more comorbidities and patient polypharmacy. A prospective randomized study is needed to show the protective effects of statins on gallbladder surgery.

## Competing interests

The authors declare that they have no competing interests.

## Authors’ contributions

JP revised the data, performed the statistical analysis and drafted the tables and the manuscript. He made substantial contributions to conception and design of study. HK revised manuscript and helped greatly to draft the manuscript. ME, PK revised manuscript. VK revised tables, advised the use of statistics and interpretation of data. TK, MP, LK collected data and drew up a part of the preliminary results. HP conceived of the study and participated in its design and coordination and helped to draft the manuscript and gave final approval of the version to be published. All authors read and approved the final manuscript.

## Authors’ information

JP MD and PK MD are surgeons in Kuopio University Hospital. HP and ME are Professors of surgery in Eastern Finland University and surgeons in Kuopio University Hospital. VK PhD statistic and HK MD are experts in Finland Medical Association. TK, MP and LK are medical students in Eastern Finland University.

## Pre-publication history

The pre-publication history for this paper can be accessed here:

http://www.biomedcentral.com/1471-230X/14/119/prepub
